# Laceration of the Perineum and Post Partum Hæmorrhage

**Published:** 1868-12-01

**Authors:** Hiram Wanzer

**Affiliations:** No. 172 West Twelfth Street; Chicago, Ill.


### LACERATION OF THE PERINEUM AND POST PARTUM HÆMORRHAGE.

*Two Cases reported to the Chicago Medical Society,*

BY HIRAM WANZER, M.D., CHICAGO, ILL.

CASE NO. 1.— I was called to Mrs. B., æt. 28, August 13th last. She had been in labor with her first child twenty hours, under the care of a midwife. Upon digital examination, I found the head presenting at the superior strait, the os uteri soft and moderately dilated, the soft parts were quite unrelaxed, and there was also rigidity of the perineum. The pains up to my arrival had been energetic, but the uterine contractions failed, even after complete dilation of the os, to increase the slightest advancement of the head. After waiting some four hours longer, I applied the forceps. The difficulties were somewhat increased by the mal-position of the head, the forehead looking to the left sacro-iliac junction, and the occiput to the right; consequently the blades were applied to the forehead and occiput of the child. It required a degree of force to dislodge the head, and bring it down into the excavation. During traction she complained severely of crampings. Had I withdrawn the instruments after the head's descent, and left her to the unassisted powers of nature, in order that the perineum and soft parts might relax physiologically, delivery might possibly have been accomplished without further interference, but I was fearful that further delay would not only increase the exhaustion of the mother, but compromise the life of the child. The rapid delivery of a preternaturally large head, apparently out of proportion to the parts of the mother, caused not only the complete division of the perineum, but also the laceration of both external and internal sphincter ani muscles, together with an involvement of the recto-vaginal septum, of one and a half inches, opening the vagina and rectum into one passage. A full anodyne being given, I immediately coapted the edges of the lacerated perineum by two deep ligatures. One of them came away on the 2nd, the other on the 5th day. Granulations at this time were quite luxuriant at the bottom of the wound. She was placed upon the side, and vaginal enemas of tepid waters were daily used, and at no time did the water escape by the rectum. The bowels were kept constipated by the *Tinct. Opii* for the first ten days, when an oleaginous enema was given, inducing a free evacuation without suffering. They were used occasionally for two weeks longer, while strict recumbency was enjoined. She was at this time able to resume her household duties. Her diet consisted of fluid nourishment; such as could be absorbed into the blood with as little fæcal residue as possible. The six weeks following parturition, she complained of want of power or contractility in the sphincters, to restrain the fæces, but since that time she states that she can hold them indefinitely; showing the ultimate possibility of their tonicity being re-established. Both the mother and child have done well. I would ask the experience of the society in laceration of the perineum, caused by the maladroit use of instruments, or otherwise, and their method of procedure.

Case No. 2.—I was called Sunday, August 30th last, to Mrs. C., æt. 31, at 7½ A.M. She had been in labor with her seventh child since 2 o'clock that morning. She stated that the pains had been quite energetic up to the time of my arrival. Upon examination, the os uteri was fully dilated, the vertex presenting, the soft parts well relaxed, and in less than three-quarters of an hour the child was born. I waited a few moments, and proceeded to the delivery of the placenta. Upon gentle traction of the cord there was some hæmorrhage, not sufficient to cause alarm; there had been none during labor. After the delivery of the after-birth, gentle frictions over the uterus were applied, until sufficient contractility of the organ was insured. The adjustment of a moderately tight bandage was not neglected. Upon leaving the patient, I requested them to summon me immediately should any thing occur unfavorably; otherwise I would call the next day. In about an hour a most alarming and exhausting hæmorrhage supervened. The patient, in a few hours' time, had been reduced from full health and plethora, to the sunken eye, the palor, the collapsed and cold surface, and the almost pulseless condition we find in Asiatic cholera. The blood clots from the mouth of the uterus and vagina were immediately removed; the pillows taken from under the head; the foot of the bed was elevated to an angle of about thirty degrees. Pressure and friction were applied over the uterus until the organ was felt to contract sufficiently; also cold applications to the vulva, warmth to the feet, and re-adjustment of the bandage. Two drachms of *Tinct. Ergot* was given every hour, with perseverence in *position*, and perfect quietude and recumbency. The hæmorrhage, in a short time, was completely arrested, and she entered upon convalescence. She states she had suffered from post-partum hæmorrhage at her three last accouchments, and in one instance had hæmorrhage before labor, but never to an alarming extent. As there was no complication apparent in the case, I can not ascribe cause for the accident. Nothing abnormal had transpired during gestation or the delivery. I regard *position* our sheet-anchor in these alarming cases; but for it the mother might have perished. I view all other agents simply valuable auxiliaries in post-partum and in other exhausting hæmorrhages.

No. 172 West Twelfth Street.

